# αADα Hybrids of *Cryptococcus neoformans*: Evidence of Same-Sex Mating in Nature and Hybrid Fitness

**DOI:** 10.1371/journal.pgen.0030186

**Published:** 2007-10-19

**Authors:** Xiaorong Lin, Anastasia P Litvintseva, Kirsten Nielsen, Sweta Patel, Anna Floyd, Thomas G Mitchell, Joseph Heitman

**Affiliations:** Department of Molecular Genetics and Microbiology, Duke University Medical Center, Durham, North Carolina, United States of America; Washington University, United States of America

## Abstract

Cryptococcus neoformans is a ubiquitous human fungal pathogen that causes meningoencephalitis in predominantly immunocompromised hosts. The fungus is typically haploid, and sexual reproduction involves two individuals with opposite mating types/sexes, **α** and **a**. However, the overwhelming predominance of mating type (*MAT*) **α** over **a** in C. neoformans populations limits **α**–**a** mating in nature. Recently it was discovered that C. neoformans can undergo same-sex mating under laboratory conditions, especially between **α** isolates. Whether same-sex mating occurs in nature and contributes to the current population structure was unknown. In this study, natural **α**AD**α** hybrids that arose by fusion between two **α** cells of different serotypes (A and D) were identified and characterized, providing definitive evidence that same-sex mating occurs naturally. A novel truncated allele of the mating-type-specific cell identity determinant *SXI1**α*** was also identified as a genetic factor likely involved in this process. In addition, laboratory-constructed **α**AD**α **strains exhibited hybrid vigor both in vitro and in vivo, providing a plausible explanation for their relative abundance in nature despite the fact that AD hybrids are inefficient in meiosis/sporulation and are trapped in the diploid state. These findings provide insights on the origins, genetic mechanisms, and fitness impact of unisexual hybridization in the *Cryptococcus* population.

## Author Summary

Cryptococcus neoformans is a major cause of fungal meningitis, predominantly in immunocompromised individuals. This fungus has two mating types/sexes, **a** and α, and mating typically requires two individuals with opposite mating types. It is mysterious why the α mating type is overwhelmingly predominant in nature and how the capacity for sexual reproduction is maintained in a largely unisexual population. We postulated that same-sex mating between α isolates may occur naturally, as it does under laboratory conditions. By analyzing natural *Cryptococcus* diploid hybrid isolates containing two α alleles of different serotypic origins, this study demonstrates that same-sex mating transpires in nature. The observations that Sxi1α, a sex regulator encoded by the mating type locus, is frequently altered in C. neoformans hybrids but rarely in the haploid population, and that Sxi1α is also altered in the fertile VGIII group of the sibling species C. gattii by a different mutation support the hypothesis that these *SXI1α* mutations may enhance fertility, possibly in concert with other genomic changes. Our study provides insights on the genetic and environmental factors that play important roles in the evolution of the current population structure of this pathogenic fungus.

## Introduction

The level of genetic variation within a species is correlated with evolutionary potential [1]. Hybridization can provide genetic variation within and between populations by yielding progeny more fit in novel or changing environments, and both intra- and interspecies hybridization are a driving force for evolution [2,3]. Hybridization is observed in animals, and is especially common in plants [4–8]. Hybrids also occur in microorganisms. For example, Trypanosoma cruzi, the cause of Chagas disease, descends from two ancestral hybridization events [9,10]; influenza viruses undergo antigenic variations and host range shifts through hybridization and reassortment [11]; and in the parasite *Leishmania*, which has no known sexual cycle and a largely clonal population structure, recombinant strains can be generated through interspecific hybridization [12–15].

Because of their morphological and genomic plasticity, fungi are subject to profound genetic changes, including those resulting from hybridization. Indeed, hybridization is one of the most significant biological forces driving fungal evolution, as illustrated by the *Saccharomyces* sensu stricto complex [16]. This species complex descends from an ancient whole genome duplication event in which two related yeast species hybridized ∼100 million years ago [17–20]. Hybridization can confer novel features; for example, *S. cerevisiae–S. paradoxus* hybrids exhibit thermal stress vigor [21]. In plant fungal pathogens, hybridization produces novel physiological traits including enhanced virulence [22–24]. By comparison, less is known about the impact of hybridization on the virulence of human pathogenic fungi.

Cryptococcus neoformans is a cosmopolitan human fungal pathogen that causes meningoencephalitis in predominantly immunocompromised hosts [25]. Cryptococcal meningitis is the most common fungal infection of the central nervous system and is considered an AIDS defining condition [25–29]. This species is classified into three serotypes based on capsular agglutination reactions [30]: serotype A (C. neoformans var. *grubii*, mostly haploid), serotype D (C. neoformans var. *neoformans*, mostly haploid), and AD hybrids (mostly diploid). Serotype A is responsible for the vast majority of human infection (95% worldwide) [25], but AD hybrids can be fairly common, especially in Europe (∼5%–30%) [31–37], and are likely more common than currently appreciated [32,37–39].

**Figure 1:**
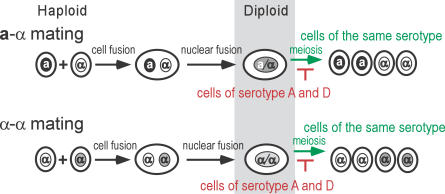
C. neoformans Mating Cycles During **a–**α mating (above), haploid **a** and α cells undergo cell–cell fusion to produce dikaryotic filaments, and nuclear fusion occurs late in the basidium to produce a transient diploid **a**/α state, which immediately undergoes meiosis and sporulation. Serotype-compatible parental strains produce viable haploid basidiospores of **a** and α mating type [43,44]. During same-sex mating (below), haploid cells of one mating type (mostly α) become diploid cells (α/α), which can undergo meiosis and sporulation (lower panel) [40,48]. In either mating cycle, if the parental strains are of different serotypes, hybrids are trapped in the diploid state because of an impaired ability to undergo meiosis as a consequence of DNA divergence (5%–10% nucleotide polymorphisms between serotype A and D). Outer circles represent cells and inner circles represent nuclei; **a** or α indicate mating type. Green indicates efficient meiosis, and red indicates impaired meiosis.

Because this fungus is ubiquitous in nature, and humans are infected through inhalation of infectious propagules from the environment [40–42], it is important to understand the natural life cycle and its impact on population structure. The bipolar mating system of C. neoformans has been well-defined under laboratory conditions. Mating involves cell–cell fusion of haploids of opposite mating type, **a** and α, to produce dikaryotic filaments. Nuclear fusion occurs at the tip of the filaments in a fruiting body (the basidium) resulting in a transient **a**/α diploid that immediately undergoes meiosis and sporulation [43,44] (Figure 1). Because clinical and environmental isolates of C. neoformans are predominantly of α mating type (>98%–99.9%) [25,45], it is difficult to envision that **a–**α mating is the only significant means by which genetic diversity is generated in nature. C. neoformans serotype D strains undergo monokaryotic fruiting to produce filaments and basidiospores under laboratory conditions [46,47]. Fruiting was recently recognized to be a modified form of sexual reproduction occurring between strains of the same mating type [48] (Figure 1). Because monokaryotic fruiting is commonly observed in serotype D α isolates [46–48], and the *MATα* allele enhances fruiting under laboratory conditions [49], same-sex mating could significantly impact the population structure of this pathogenic fungus in nature.

Although the global population of C. neoformans is largely clonal, recombination does occur at a low level [50–54]. Recently, phylogenetic analysis of the sibling species C. gattii has shown that same-sex mating between two different α strains may have given rise to a more virulent strain occupying a new environmental niche and causing the Vancouver Island outbreak [55]. Population genetic studies of C. neoformans serotype A veterinary isolates in Sydney, Australia, also reveal evidence of recombination in a unisexual α population, providing further indirect support for the occurrence of same-sex mating in natural populations (D. Carter, personal communication). In this study, characterization of natural diploid αADα hybrids provides definitive evidence for same-sex mating occurring in nature.

Under laboratory conditions, intervarietal matings between strains of serotype A and D lead to cell–cell fusion, but genetic differences between these divergent serotypes (∼5%–10% nucleotide polymorphisms) severely limits meiosis and thus few, if any, viable haploid basidiospores are produced [56,57]. Consequently, most natural AD hybrids remain in the diploid (or aneuploid) state (Figure 1), and analysis of these AD hybrids can reveal the genomic nature of their parental strains. For example, most reported AD hybrids are αAD**a** or **a**ADα (mating type/serotype–serotype/mating type) [32–36,57,58], reflecting their origin from traditional **a–**α mating between serotype A and D strains. All extant **a**ADα hybrids appear to derive from a cross between African serotype A strains (A**a**) and serotype D strains (Dα) followed by clonal expansion and emigration from sub-Saharan Africa, the only region where serotype A isolates of** a** mating type are common [53,59].

In this study, we identified and characterized natural αADα hybrids that arose from same-sex mating between two α strains of A and D serotypes, providing definitive evidence that the laboratory-defined same-sex mating process occurs in nature. In addition, our analysis reveals a common feature in all **a**ADα and αADα hybrids tested: a C-terminal deletion in the serotype D *SXI1α* gene located in the *MAT* locus, which encodes a homeodomain transcription factor regulating mating [60]. Characterization of populations containing the C-terminally truncated *SXI1α* serotype D (*SXI1Dα*) allele suggests that this mutation may have contributed to the origin of AD hybrids.

The common presence of AD hybrids in both clinical and environmental samples may be indicative of hybrid vigor [33,35,61]. However, unlike clearly documented cases of increased fitness and epidemiological success of plant-pathogenic fungal hybrids [23,62–65], examples of hybrid fitness in human pathogenic fungi have not been well-documented. Previous studies of C. neoformans AD hybrids revealed variable virulence potential [57,58,66,67]. This ambiguity may be due to the analysis of genetically diverse αAD**a** and **a**ADα isolates, which exhibit considerable phenotypic and genotypic variation. The presence of both **a** and α mating types in a diploid strain may also complicate virulence studies if pheromone sensing occurs during infection [68–70]. Here, αADα hybrids were constructed in defined genetic backgrounds and analyzed for hybrid fitness and virulence. In vitro, laboratory-constructed αADα hybrids exhibited hybrid vigor, and were more UV- and temperature-resistant than either parent. Other virulence attributes of the αADα hybrid were similar to (e.g., capsule) or intermediate between (e.g., melanin) those of the parents. In a murine inhalation model, the laboratory-constructed αADα hybrid exhibited virulence similar to that of the serotype A parent. These observations demonstrate benefits of hybridization in C. neoformans, which may enable less robust serotype D strains to survive both during infection and in the environment.

## Results

### Identification of Natural αADα Diploid Hybrids

**Figure 2:**
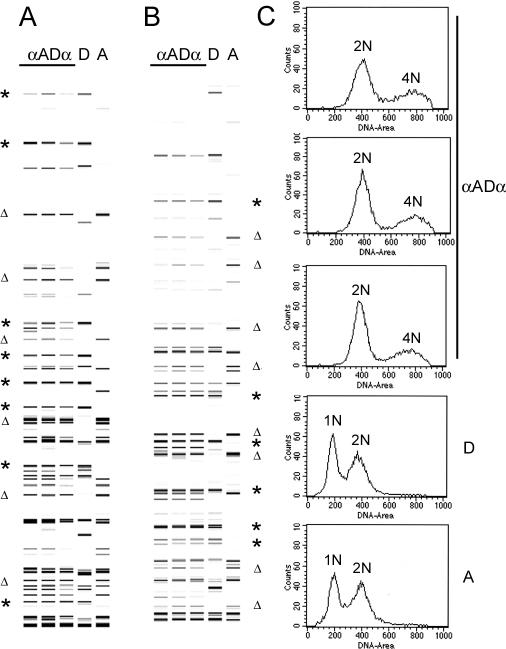
Environmental Isolates Are Diploid AD Hybrids (A and B) Environmental isolates 5–19, 6–20, 42–10, and serotype A (H99, Aα) and D (JEC21, Dα) control strains were analyzed by AFLP analysis using primer sets EAC (A) and ETG (B) as previously described [53,68]. Asterisks indicate serotype D–specific products, and triangles indicate serotype A–specific products. Environmental isolates 5–19, 6–20, 42–10 displayed a superimposition of the serotype A and D AFLP patterns, indicative of their AD hybrid nature. (C) Flow cytometry profiles of the environmental isolates 5–19, 6–20, 42–10, and the haploid control strains JEC21 and H99 after staining with the fluorescent dye propidium iodide. 1N, 2N, and 4N indicate nuclear content. The *x-*axis indicates fluorescence intensity reflecting the DNA content, and the *y-*axis indicates cell counts.

A report from Litvintseva et al. in 2005 indicated the potential existence of environmental αADα hybrids isolated from North Carolina, USA [35]. To ensure these were indeed AD hybrids, three such isolates and control strains were analyzed by amplified fragment length polymorphism (AFLP) analysis. AFLP results using two primer pairs showed that all three isolates generated a banding pattern representing a composite between those of serotype A and D strains, indicative of an AD hybrid (Figure 2A and 2B). These strains also contained twice the cellular DNA content of haploid controls based on fluorescence flow cytometry analysis (Figure 2C), and are therefore diploid.

Based on serotype- and mating-type-specific PCR, all three isolates have serotype A– and serotype D–specific genes, both within the mating type locus (*MAT*) and in other genomic regions (Table 1), further confirming their AD hybrid nature. Sequence analysis suggested the three isolates could be clonal, as PCR-amplified gene sequences were identical (data not shown). The combined sequences for five different serotype A–specific genes (*STE20α*, *SXI1α*, *GPA1*, *CNA1*, and *PAK1*) were 99.9% identical to those of the sequenced serotype A reference strain H99 (4,226/4,230 bp) [71]. The sequences for four different serotype D–specific genes (*STE20α*, *GPA1*, *CNA1*, and *PAK1*) were 99.86% identical to those of JEC21 (2,886/2,890 bp), a sequenced serotype D reference strain [72]. Because these AD hybrid isolates contain α mating type genes from both serotype A (*STE20α* and *SXI1α*) and D (*STE20α*) and lack **a** mating type genes of either serotype (Table 1), they are αADα strains that originated from two α parental strains of serotype A and D. This provides the first direct evidence, to our knowledge, of the cell–cell fusion step of same-sex mating occurring in nature.

**Table 1:**
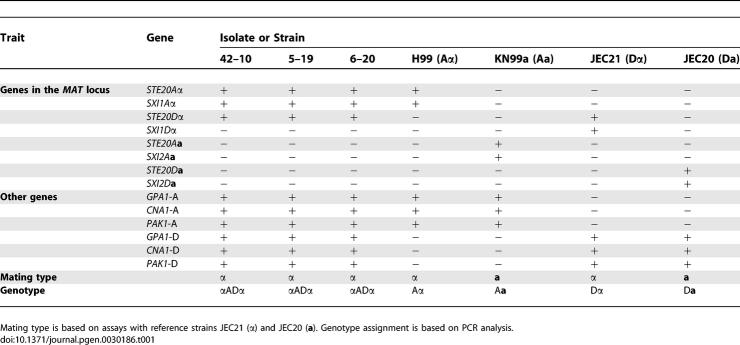
North Carolina Environmental AD Hybrids are αADα

### αADα Hybrids Mate as α

**Figure 3:**
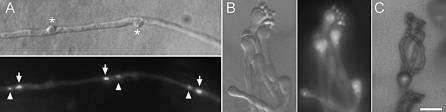
αADα Hybrids Mate as α Cells (A) Hyphae produced by mating between the natural αADα hybrid 6–20 and JEC20 reference strain on V8 medium were fixed and stained with DAPI. Diploid nuclei (arrows) are brighter and larger than their haploid counterparts (arrowheads) when stained with DNA fluorescent dyes [73,97]. Dikaryotic mating hyphae containing two nuclei per compartment, which alternate position at each conjugate division, were observed during mating. Fused clamp cells are indicated by asterisks in the DIC image. (B) Basidia at different stages of development. (C) Four long spore chains produced on the surface of a basidium during mating. Scale bar, 10 μm.

The mating behavior of the natural αADα hybrids was examined in crosses with the reference strains JEC20 (**a**) and JEC21 (α). The αADα hybrids mated with the **a** reference strain JEC20 to produce mating dikaryotic hyphae with clamp connections (Figure 3), and did not mate with the α reference strain (data not shown). The two parental nuclei (diploid α/α and haploid **a**) alternated positions in adjacent hyphal cells, a hallmark of compatible matings in basidiomycetous fungi [48,73]. Basidial fruiting bodies were also observed in different developmental stages, they contained one or multiple nuclei, and some were decorated with four long chains of spores (Figure 3). These morphological characteristics are similar to those of matings between haploid α and **a** cells. However, despite apparently normal morphological differentiation, the spores generated were not viable, and all dissected spores from a cross between the diploid αADα hybrid 6–20 and the **a** haploid strain JEC20 failed to germinate (*n* = 105), indicative of abnormal meiosis, as expected from a triploid. Our observations indicate that the αADα hybrid mates as α, but is unable to complete the final stages of sexual reproduction, including spore germination.

### αADα Hybrids Contain the α Mating Type Locus from Both Serotype A and D

Because the *SXI1Dα* allele could not be amplified from the αADα hybrids with the primers tested (Table 1), the mating type locus of the αADα hybrids was further analyzed to ascertain whether any genetic alterations were apparent. The *MAT* locus of C. neoformans is unusually large (>100 kb) compared to most fungi and encodes more than 20 proteins [74]. Because of the complex nature of the *C. neoformans MAT* locus, all genes within the *MAT* locus of the natural αADα hybrid were examined by comparative genome hybridization (CGH).

**Figure 4:**
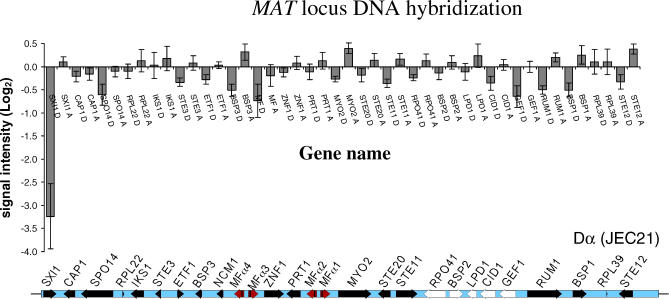
CGH of the αADα Hybrid Mating Type Locus Fragmented genomic DNA from the environmental isolate 6–20 and a mixture of genomic DNA from strains H99 (Aα) and JEC21 (Dα) was labeled with fluorescent dyes and competitively hybridized to a 70-mer genomic array. The fluorescent signal level was normalized across the genome, and the average of six independent replicates of the fluorescent intensity ratio for the serotype A and D *MAT* locus α alleles is shown. Error bars represent the standard deviation. “A” or “D” at the end of each gene name indicates the serotype A– or serotype D–specific allele. A schematic representation of the mating type locus is illustrated below. Blue indicates intergenic regions, red indicates the pheromone gene cluster, and white indicates highly conserved genes [74].

Mating-type- (**a** and α) and serotype-specific (serotype A and D and C. gattii) 70-mer probes for all genes in the *MAT* locus (Aα, A**a**, Dα, and D**a** alleles for each *MAT* gene) were designed previously for microarray analysis [49]. Here genomic DNA was labeled and hybridized to microarray slides to characterize the mating type locus gene content. Genomic DNA of the natural αADα hybrid 6–20 and the control (a mixture of H99 [Aα] and JEC21 [Dα]) were labeled with fluorescent dyes and competitively hybridized to a genomic microarray slide containing the mating-type- and serotype-specific 70-mers. The log_2_ ratio of fluorescence intensity between the hybrid and the control for all **a** genes was close to zero regardless of serotypes (the average log_2_ ratio of fluorescence intensity was within ± 0.4, meaning that the fold difference between hybrid and control fell into the range of 0.76∼1.32; data not shown), indicating the genetic contents of the control, and sample were similar. Because there were no a genes in the control, this showed that **a** genes were also absent in the hybrid strain, consistent with the PCR analysis (Table 1). To ensure that lack of hybridization to A**a** or D**a** probes was not due to failure of the a 70-mers on the microarray slides, hybridizations of Aα/D**a**, A**a**/Dα, and A**a**/D**a** samples using genomic DNA from reference strains were performed. The A**a** and D**a** probes were functional based on this analysis (Figure S1). As shown in Figure 4, the overall fluorescence intensity of α genes in the *MAT* locus from the natural hybrid isolate 6–20 and the control (Aα + Dα) was similar for both the serotype A and D alleles (log_2_ ratio of fluorescence intensity was within ± 0.5, meaning that the fold difference between hybrid and control fell in the range of 0.71∼1.41). The only exception was that the *SXI1Dα* allele appeared to be missing in the natural αADα hybrid, as the fluorescence intensity of the hybrid *SXI1Dα* was much lower than that of the control (log_2_ hybrid/control = −3.24, which means hybrid/control ≈ 0.1). This CGH result is consistent with the *SXI1α* PCR analysis (Table 1), indicating that hybrid 6–20 contains all α genes from both serotype A and D with the apparent exception of the *SXI1Dα* allele.

However, because the array used was not a tiling array, other potential mutations in the mating type locus, such as indels in regions not covered by the probes and single nucleotide alterations, might not be detected.

### αADα Hybrid *SXI1Dα* Allele Is C-Terminally Truncated

**Figure 5:**
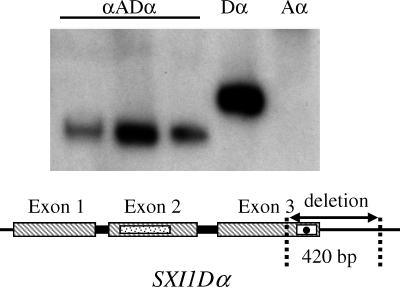
The *SXI1Dα* Gene Bears a C-Terminal Deletion in the αADα Hybrids Genomic DNA of environmental isolates 5–19, 6–20, and 42–10, and reference strains JEC21 (Dα) and H99 (Aα), was digested with NcoI and probed with the *SXI1Dα* ORF (above). Weak cross-hybridization to *SXI1Aα* at higher molecular weight in AD hybrids and the serotype A control strain, but not in the serotype D control strain, is not shown here. Structure of the truncated allele of the *SXI1Dα* gene in the three environmental isolates 5–19, 6–20, and 42–10 (below). The black line indicates other genomic DNA, boxes with diagonal lines indicate exons, thick black lines indicate introns, the box with dots indicates the homeodomain, and dashed lines mark the 420-bp deletion in the C-terminus of the ORF (exon 3) and part of the 3′ untranslated region. The open box indicates the location of the *SXI1Dα* oligomer on the array. The dot inside the open box indicates the location of primer JOHE15636 used to amplify the *SXI1Dα* gene in the PCR screening.

Because the *SXI1Dα* gene in the *MAT* locus of the αADα hybrids did not amplify using *SXI1Dα*-gene-specific primers (Table 1), or yield a hybridization signal during CGH analysis (Figure 4), the structure of the *SXI1Dα* locus in the natural αADα hybrids was examined by Southern hybridization. Surprisingly, hybridization to the *SXI1Dα* ORF probe was observed, but the size of the hybridizing band was decreased for the natural αADα hybrids compared to the wild-type serotype D control, suggesting that a shorter version of the *SXI1Dα* gene was present (Figure 5). Sequencing of the *SXI1Dα* allele from the three αADα hybrids revealed a C-terminal truncation of the ORF (119 bp) and a partial deletion of the 3′ untranslated region (301 bp). Thus, the genomic locus is 420 bp shorter in the αADα hybrids (Figure 5). The 70-mer oligonucleotide on the microarray slide used to detect the *SXI1Dα* gene lies within the C-terminal deletion interval, and the sequence of one of the primers (JOHE15636) used to PCR amplify the *SXI1Dα*-specific gene was also within the missing region (Figure 5), explaining the apparent absence of the *SXI1Dα* gene in the PCR and CGH analyses (Table 1; Figure 4).

### The *SXI1Dα* Truncation Allele Is Present in All **a**ADα and αADα Hybrids Tested, but Is Uncommon in Serotype D α Haploid Isolates

To test whether the C-terminal deletion in the *SXI1Dα* gene is unique to the αADα isolates from North Carolina, or is a uniform feature of the **a**ADα and αADα hybrids with a Dα parental origin, additional hybrids were analyzed. Interestingly, all of the **a**ADα and αADα hybrid strains tested share precisely the same C-terminal truncated version of *SXI1Dα* (Table 2). Four hypotheses could explain the presence of the truncated *SXI1Dα* allele in hybrid strains. (1) The “pre-fusion fitness” model: the truncated *SXI1Dα* allele may confer an advantage to haploid serotype D strains, and selection for the shorter version of *SXI1Dα* occurred prior to cell fusion. In this model, the truncated version of *SXI1Dα* is prevalent in **a**ADα and αADα hybrids simply because it is common in the Dα population. (2) The “pre-fusion fertility” model: selection for this C-terminally truncated *SXI1Dα* was prior to cell fusion. This version of *SXI1Dα* may enhance the fertility of Dα strains and therefore is common in **a**ADα and αADα hybrid strains that result from fusion between A**a** or Aα strains and Dα strains with this allele. (3) The “post-fusion fitness” model: the *SXI1Dα* truncated version may confer an advantage to AD hybrids such that selection for this allele occurred after hybrid formation. This advantage could involve limiting sporulation, leading to fewer inviable spores in AD hybrid strains. (4) The “natural variant” model: this *SXI1Dα* allele is a neutral variant that confers no selective benefit.

**Table 2:**
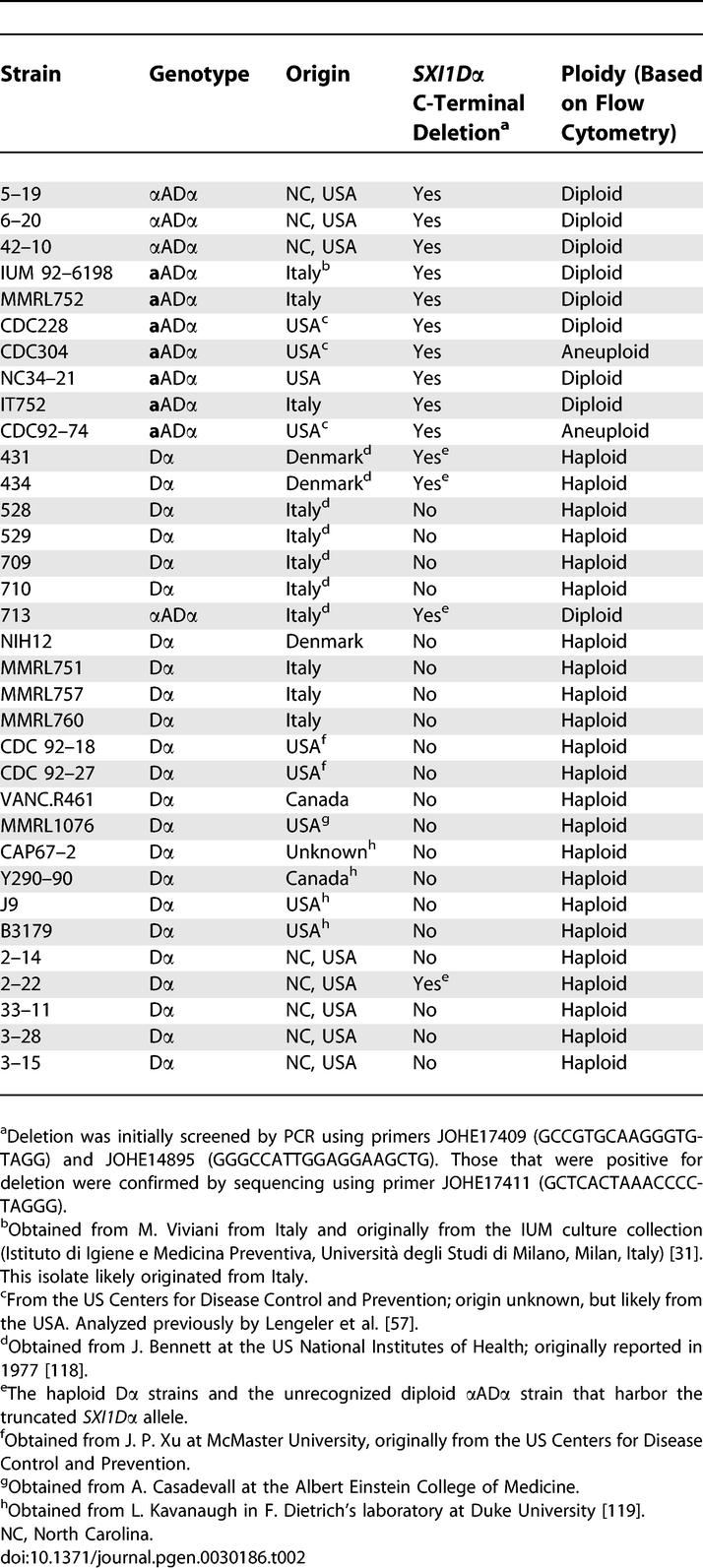
Polymorphism of *SXI1Dα* in Hybrid and Haploid Dα Populations

To test these hypotheses, the prevalence of the C-terminally truncated version of the *SXI1Dα* allele was investigated in natural Dα isolates. If selection for this *SXI1Dα* allele occurred prior to the cell fusion events that produced AD hybrids (“pre-fusion fitness” and “pre-fusion fertility” models), this allele should be present in the serotype D α haploid population. If selection for this allele occurred after cell fusion, then it would be expected to be absent in the Dα population (“post-fusion fitness” model). If there was no selection, then this allele need not be common in either the hybrid or the Dα population (“natural variant” model).

Twenty-four isolates recorded as serotype D α strains were screened by PCR to detect *SXI1Dα* size polymorphisms, and four isolates were found to contain the truncated allele, while the remaining 20 isolates contain the wild-type allele (Table 2). The truncated version of *SXI1Dα* in these four isolates was sequenced, and the deletion site was identical to that found in the **a**ADα and αADα hybrids. Interestingly, one of five North Carolina Dα isolates, each representing a different genotype [35], harbors the C-terminally truncated *SXI1Dα* allele. These five Dα strains were isolated together with the natural αADα hybrids in a previous study [35]. The North Carolina αADα hybrids bearing the C-terminally truncated *SXI1Dα* allele represent the common AD genotype (76%, or 41/54) in this region [35], further supporting the hypothesis that selection for this allele could have occurred. To ensure that these isolates are indeed haploid Dα strains and not unrecognized hybrids, ploidy was analyzed by fluorescence flow cytometry. As shown in Table 2, with one exception (isolate 713), all of the serotype D isolates tested were haploid. Isolate 713 showed a diploid nuclear DNA content and was found to be an unrecognized αADα hybrid isolate from Italy (see below).

Thus, the truncated *SXI1Dα* allele is present in the global natural serotype D α isolates, albeit at a relatively low level (∼13%, or 3/23), which does not support the “pre-fusion fitness” or “post-fusion fitness” models. The truncated *SXI1Dα* allele is uniformly present in the **a**ADα and αADα hybrid population (100%, or 10/10) (Table 2), which supports the “pre-fusion fertility” or “post-fusion fitness” models. All strains with the truncated *SXI1Dα* allele harbor an identical *SXI1Dα* allele, while those strains without the truncation harbor distinct *SXI1Dα* alleles based on the sequence of the *SXI1Dα* 5′ region preceding the deletion site. Thus, the novel truncated allele likely arose once in the haploid progenitor population, arguing against the “post-fusion fitness” selection model. These findings support the “pre-fusion fertility” model, in which the *SXI1α* truncation allele enhances fertility of the serotype D α haploid parental progenitors and, as a result, increases fusion with an A**a** or Aα partner to yield the **a**ADα and αADα hybrid populations.

During this screening, another αADα hybrid isolate (713) was identified. This diploid isolate contains α mating type genes from both serotype A (*STE20Aα* and *SXI1Aα*) and D (*STE20Dα*), lacks **a** mating type gene of either serotype type based on mating-type- and serotype-specific PCR (data not shown), and is an unrecognized αADα hybrid. To confirm this, the *MAT* locus was characterized by CGH. Isolate 713 displayed a CGH *MAT* profile similar to that of the αADα hybrids from North Carolina (Figure S2). All of the mating type genes of both the Aα and the Dα alleles were similar to the control (Aα + Dα) with the only exception being the *SXI1Dα* gene, which was also truncated in this natural αADα hybrid. The discovery of an independent αADα isolate from Italy suggests that same-sex mating is not restricted geographically, consistent with the fact that Aα and Dα isolates are globally distributed worldwide in nature and are often sympatric. However, we cannot exclude that an ancestral αADα isolate clonally expanded to distinct locations.

### *SXI1α* Gene Harbors C-Terminal Premature Stop Codons in the C. gattii VGIII Lineage

To investigate if altered *SXI1α* alleles also occur in other members of the *Cryptococcus* species complex, known sequences of the *SXI1α* gene in the sibling species C. gattii were analyzed [55]. C. gattii and C. neoformans diverged from a common ancestor ∼37 million years ago and are recognized to be separate species [75]. C. gattii is divided into four molecular types: VGI, VGII, VGIII, and VGIV [40]. Although the majority of C. gattii strains are sterile, a significant proportion of VGIII isolates are fertile [55,76]. The *SXI1α* gene sequences in strains of the three molecular types, VGI (10/10), VGIV (4/4), and VGII (10/11) appeared wild-type (data not shown) with the exception of one VGII strain (WM178, 1/11) in which the *SXI1α* gene contains a frameshift mutation (Figure S3). Interestingly, a premature stop codon is present in the C-terminus of the *SXI1α* gene in the majority of strains of the VGIII molecular type (7/8, or 87.5%) (Figure S3). This stop codon truncates the C-terminus of Sxi1α (corresponding to residue 358 in Sxi1Dα) seven amino acids N-terminal to the deletion site found in the truncated *SXI1Dα* allele in the **a**ADα and αADα populations (365 aa) (Figure S3). Importantly, the homeodomain (aa 144–205 in Sxi1Dα) [60] is intact in both truncated *SXI1α* alleles. The observation of two different mechanisms of C-terminal truncation in the *SXI1α* gene occurring in subgroups of two different species (C-terminal deletion in C. neoformans serotype D and AD hybrid strains and premature stop codon in VGIII C. gattii isolates) indicates that C-terminally truncated versions of the *SXI1α* gene have arisen independently at least twice in the *Cryptococcus* species complex.

### *SXI1α* C-Terminally Truncated Alleles Are Functional

**Figure 6:**
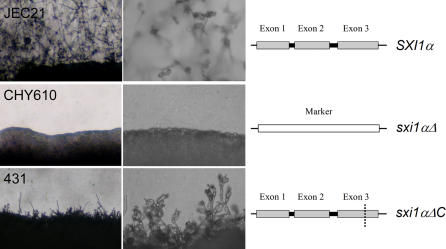
Haploid Dα Strains Bearing the *SXI1Dα* C-Terminal Deletion Are Fertile Natural Dα isolates with the C-terminal deletion in the *SXI1Dα* gene mate and produce mating hyphae, basidia, and spores. From top to bottom are wild-type α reference strain JEC21, isogenic *sxi1Dα*Δ mutant strain CHY610, and nonisogenic natural serotype D isolate 431 with the *SXI1Dα* C-terminal deletion, all mated with the reference **a** strain JEC20. Mating filaments are shown in the left images (×40), and basidia with spore chains are shown in the right images (×200). Diagrams at far right indicate the *SXI1Dα* allele*.*

The *SXI1α* gene is a master regulator of sexual reproduction [60]. Deletion of this gene does not prevent cell–cell fusion, but blocks further sexual morphological differentiation into dikaryotic hyphae, meiosis, and development of basidiospores during **a**–α mating [60]. The C-terminal deletion of the *SXI1Dα* gene does not prevent sexual differentiation during mating based on the fact that the two natural Dα strains (431 and 434) with a C-terminal deletion in the *SXI1α* gene still produce mating hyphae and abundant basidiospores when crossed with the reference strain JEC20 (Figure 6). Differences in filamentation and sporulation observed between the wild-type strain JEC21 and the nonisogenic natural strain 431 could be attributable to other genetic differences. Spores dissected from a cross between strain 431 and JEC20 were viable (germination rate = 83%, *n* = 72) and showed a typical 1:1 Mendelian segregation of mating types (**a**:α = 31:29), indicative of normal meiosis. An engineered strain in the JEC21 background with the C-terminally truncated allele of *SXI1Dα* replacing the wild-type *SXI1α* allele also mated like wild-type, indicating the truncated *SXI1α* allele is functional (Figure S4). These observations indicate that the C-terminal deletion of the *SXI1α* gene does not impair morphological development or meiosis during mating.

**Figure 7:**
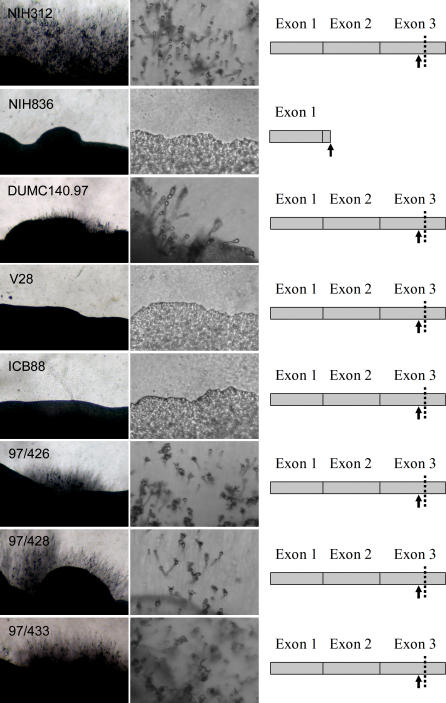
C. gattii Strains with a Premature C-Terminal Stop Codon in the *SXI1α* Gene Are Fertile Natural α C. gattii VGIII isolates with variations in the C-terminus of the *SXI1α* gene mate with reference strain JEC20 to form mating hyphae and spores. From top to bottom are strains NIH312, NIH836, DUMC140.97, V28, ICB88, 97/426, 97/428, and 97/433. Mating filaments are shown in the left images (×40), and basidia with spores are shown in the right images (×200). Diagrams at far right show the corresponding *SXI1α* alleles, the location of the stop codon (arrow), and the location of the corresponding deletion site of the truncated *SXI1Dα* allele (dashed line).

The *C. gattii SXI1α* gene with the premature stop codon at the C-terminus is also functional. Five out of seven VGIII C. gattii strains that contain this *SXI1α* variant mated with the reference strain JEC20 to form mating hyphae and spores (Figure 7). Two VGIII isolates were sterile and likely harbor other unlinked mutations that impair fertility (Figure 7). The C. gattii isolate NIH836 likely harbors a nonfunctional *SXI1α* gene as an early stop codon occurs after one-third of the coding sequence (Figure S3); this isolate was sterile, consistent with the known essential role of *SXI1α* in mating [60]. Many natural C. gattii strains are sterile under laboratory conditions [77], whereas the VGIII molecular type contains many of the known fertile C. gattii isolates. Isolate NIH312, the most fertile C. gattii strain identified thus far [77], is a member of this group and harbors the *SXI1α* premature stop codon allele. These findings provide further evidence that changes in the C-terminus of the *SXI1α* gene may enhance fertility.

### Laboratory-Generated αADα Hybrid Strains Exhibit Hybrid Vigor In Vitro

Previous reports on the virulence of AD hybrids present differing results [57,58,67]. Reduced virulence of AD hybrid isolates compared to the Aα H99 reference strain was observed by Lengeler et al. [57], virulence of AD hybrids similar to that of H99 was reported by Chaturvedi et al. [58], and virulence of AD hybrids intermediate between Aα H99 and D**a** JEC20 reference strains was presented by Barchiesi et al. [67]. This variation is likely due to both different experimental models and analysis of divergent αAD**a** and **a**ADα isolates, as these isolates differ genotypically and phenotypically. The presence of opposite mating types, **a** and α, in diploid strains may also have complicated earlier virulence studies, as pheromone production and sensing may occur during infection [68–70].

To avoid these potential complications in virulence studies, AD hybrid strains of only α mating type were constructed based on the H99 (haploid Aα) and JEC21 (haploid Dα) backgrounds (see Materials and Methods for details). Both parental strains have completed genome sequences and are widely used for genetic and pathogenesis studies [72,78,79] (http://cneo.genetics.duke.edu/; http://www.broad.mit.edu/annotation/genome/cryptococcus_neoformans/Home.html).

The laboratory-generated αADα hybrid was first tested in vitro. As an environmental pathogen, C. neoformans may have evolved and maintained virulence traits through selective pressure in the environment [25,56,80,81]. Defined C. neoformans virulence factors include melanization, capsule production, and the ability to grow at high temperature, all of which confer survival advantages in both animal hosts and the environment. The ability to grow at high temperature (37–39 °C) enables human infection [82–84]; production of a polysaccharide capsule inhibits host immune responses during infection and protects cells from dehydration in the environment [85–88]; production of melanin provides protection from toxic free radicals generated by host defenses during infection and from UV irradiation in the environment [89,90]. These virulence properties enable C. neoformans and its sibling species C. gattii to be the only two highly successful mammalian pathogens in the genus *Cryptococcus* [40,56,91].

**Figure 8:**
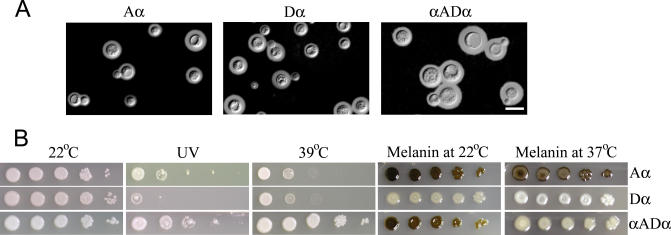
In Vitro Assays of Virulence Attributes Serotype A (H99, Aα) and D (JEC21, Dα) reference stains and the isogenic laboratory-constructed AD hybrid (XL1462, αADα) were grown in liquid YPD medium overnight and washed three times with distilled water. Cell concentration was determined by optical density at 600 nm. Cells were then incubated on DMEM for 3 d and examined microscopically with India ink to reveal capsules (A). Three microliters of serial dilutions (10×) of cells (from overnight cultures in YPD) at the same concentration were spotted onto the indicated media for phenotypic characterization (B). Cells were grown on YPD medium at 22 °C as controls for growth (first column). Cells on YPD medium were subjected to UV irradiation for 12 s (∼48 mJ/cm^2^) and then incubated at 22 °C (second column). Cells were grown on YPD medium at 39 °C (third column). Cells were grown on L-DOPA medium at 22 °C (fourth column) or 37 °C (fifth column) to assay melanin pigmentation.

In vitro virulence attributes of the laboratory-constructed hybrid strain were compared to those of the parental strains. Haploid Aα (H99), haploid Dα (JEC21), and the laboratory-constructed hybrid αADα (XL1462) strains were examined for sensitivity to UV irradiation, growth at high temperature (39 °C), capsule production, and melanization (see Materials and Methods for details). Each cell type was capable of capsule production based on microscopic observations (Figure 8A). The diploid αADα hybrid cells were larger than those of the parental Aα and Dα strains, and this was confirmed by forward scatter flow cytometry (data not shown). An association of higher ploidy with larger cell size has also been observed in other organisms [92–94]. The Aα strain H99 was more resistant to UV irradiation than the Dα strain JEC21, and the αADα hybrid strain was even more resistant to UV irradiation than the Aα parental strain (Figure 8B). Both higher ploidy, which resulted from hybridization, and the interaction of the serotype A and D genomes independently contribute to this enhanced resistance of AD hybrids to UV irradiation, based on the observation that diploid cells (αAAα or αDDα) were modestly more UV-resistant than haploid cells (Aα or Dα), but less UV-resistant than αADα hybrids (Figure S5). The αADα hybrid strain also grew significantly better at 39 °C than the Aα and Dα haploid parental strains, again displaying hybrid vigor (Figure 8B).

C. neoformans can produce melanin by oxidizing a variety of diphenolic substrates, including the neurotransmitter L-dihydroxyphenylalanine (L-DOPA) [89]. Variation in the rate of melanization yields pigmentation differences. At 22 °C, both the Aα strain and the αADα hybrid were heavily melanized compared to the Dα strain (Figure 8B). At 37 °C, melanization of the hybrid αADα was drastically reduced and was comparable to that of the less melanized Dα parental strain (Figure 8B). This observation indicates a complicated interaction of different virulence attributes (temperature and melanization) in the αADα hybrid.

In conclusion, the αADα hybrid strain displays hybrid vigor for some virulence factors under defined in vitro conditions, but the effect of hybridization on other virulence factors is complex.

### Laboratory-Generated αADα Hybrid Strain Is Highly Virulent

**Figure 9:**
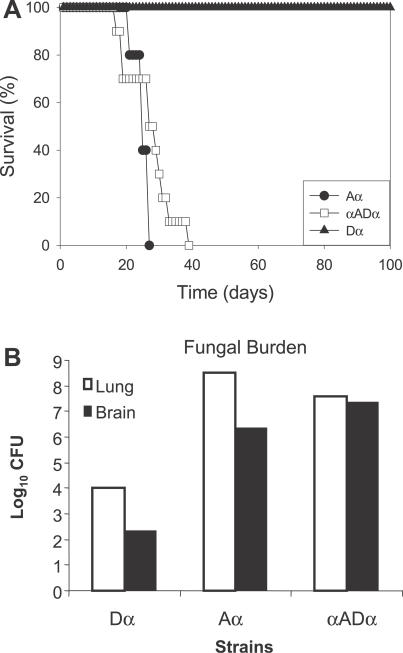
αADα Hybrid Is as Virulent as the More Virulent Serotype A Parent Serotype A (KN99α, Aα) and D (JEC21, Dα) reference stains and the isogenic laboratory-constructed αADα hybrid (XL1462, αADα) were grown in liquid YPD medium overnight at 37 °C and washed three times with PBS. Mice were infected intranasally with 5 × 10^4^ fungal cells and monitored for 100 d. (A) Survival is plotted against time after inoculation. The αADα hybrid is as virulent as the Aα parental strain (*p =* 0.371), and both the Aα and the αADα hybrid are more virulent than the Dα strain (*p =* 0.0007 and *p <* 0.0001 respectively). (B) The average number of colony-forming units (CFU) of fungal cells from the organs (lungs and brains) of two infected animals is shown.

As the effects of hybridization on in vitro virulence attributes are complex, the virulence potential of the hybrid was assayed in a murine inhalation model. Animals were intranasally infected with haploid Aα (H99), haploid Dα (JEC21), and the laboratory-constructed hybrid αADα (XL1462) strains. Animal survival and fungal burden in the lungs and brains were monitored. The αADα hybrid strain is as virulent as the highly virulent Aα parental strain, based on both survival rate (Figure 9A) (*p =* 0.371) and organ burden of fungal cells at the time of sacrifice (Figure 9B). Animals infected with the Dα strain remained viable and showed no symptoms at the conclusion of the study (day 100). Fungal burden in animals infected with the Dα strain was considerably lower than that of animals infected with the Aα or the αADα hybrid strains. This assay indicates that the Aα and αADα strains are both much more virulent than the Dα strain, and thus hybridization with an Aα partner confers a clear benefit to the less virulent serotype D α strain. Enhanced virulence in animals is not likely to be the selective pressure that gives rise to AD hybrids, as mammalian infection is not an obligate part of the normal life cycle of this environmental pathogen, but it may reflect evolved traits that contribute to the common presence of AD hybrids in nature [80,95].

## Discussion

The same-sex mating process has been observed under laboratory conditions [48] and is hypothesized to occur in nature given that C. neoformans has a largely unisexual population and the α mating type predominates in both clinical and environmental isolates. Population genetic studies also provide evidence that same-sex mating occurs in nature. For example, the Vancouver Island outbreak C. gattii strains are hypothesized to descend from two α parental strains [55], and serotype A strains from Sydney, Australia, show evidence of recombination in a unisexual α population (D. Carter, personal communication). However, direct evidence for naturally occurring same-sex mating is lacking, probably because of the difficulty of observing this process in nature.

By characterizing naturally occurring αADα hybrid strains, we present here conclusive evidence for the cell–cell fusion step in the same-sex mating process. Because of genetic divergence, hybrids have an impaired ability to undergo meiosis and remain in a diploid state where both parental genomes, including the *MAT* locus, are largely intact. These natural αADα hybrids have α mating type alleles from two parents of different serotypes that can be distinguished by serotype- and mating-type-specific PCR, CGH, and sequencing. All mating type genes (>20) of both serotype A α and serotype D α alleles are present in the AD hybrid, based on CGH, with the exception of the *SXI1Dα* gene, which bears a unique C-terminal deletion. The fact that αADα hybrids have been found in both the US and Italy suggests either that the same-sex mating process is not restricted to a specific geographic location or that αADα strains clonally expanded and dispersed. Additional AD hybrids of this nature likely remain to be recognized, as the Italian αADα hybrid strain was originally classified as a haploid Dα strain. It can be difficult to recognize αADα strains because (1) ploidy analysis of strains is not a common laboratory practice, (2) αADα hybrid strains mate as α strains in mating assays and thus do not behave like **a**ADα or αAD**a** hybrids, which are sterile or self-fertile, and (3) many AD hybrids are not recognized as hybrid strains by the serotype agglutination test commonly used in ecological and epidemiological studies [32,37–39].

Evidence has been presented to advance the hypothesis that some *MAT* homozygous isolates (α/α or **a**/**a** diploids) arise via a post-meiotic nuclear fusion event following **a–**α mating [96]. It is possible that a post-meiotic nuclear fusion event could generate **a**/**a**, **a**/α, and α/α diploid nuclei that are packaged into spores, generating *MAT* homozygous and *MAT* heterozygous diploid isolates, as originally proposed by Sia et al. [97]. However, post-meiotic nuclear fusion following **a–**α mating seems an unlikely explanation for the αADα isolates described here. First, only αADα, and no **a**AD**a,** *MAT* homozygous strains were observed. Second, the αADα isolates descend from two α parents of divergent lineages and as a consequence inherited two very divergent alleles of the *MATα* locus, in contrast to what would be expected for the post-meiotic fusion model, in which the *MATα* locus alleles would be strictly identical by descent. Third, the genetic distance between serotype A and D isolates precludes efficient meiosis and sporulation, limiting the routes by which the unusual αADα isolates could have arisen. The most parsimonious hypothesis as to the origin of the αADα diploids is same-sex mating between haploid Aα and Dα parents, and further study of the origins of other *MAT* homozygous strains (αAAα and αDDα) is warranted. We hypothesize that such isolates may also have arisen via same-sex mating, based on the findings presented here with respect to αADα isolates.

This study provides evidence for the first step in same-sex mating: cell–cell fusion. The natural conditions that stimulate cell–cell fusion events during same-sex mating are still unknown and require further investigation. Furthermore, the current study could not address meiotic reduction of the α/α diploids because none of these isolates was self-fertile under laboratory conditions. Meiosis is similarly precluded in many **a**ADα and αAD**a** hybrids. Only a minority of **a**ADα and αAD**a** hybrids were reported to be self-fertile in a previous study, and only one was observed to produce spores, which germinated poorly (<5%), reflecting a meiotic defect [57]. The extensive DNA divergence between the two serotypes likely triggers a mismatch-repair-system-evoked block to recombination, similar to that in interspecies hybrids in bacteria and budding yeasts [98–101]. In this sense, AD hybrids likely represent a genetic dead end as they cannot complete a normal sexual cycle. They are therefore a source of diversity, but not the source of diversity for the haploid population. While providing direct evidence for α–α same-sex mating in nature, the challenge remains to provide evidence for completion of the α–α sexual cycle, including meiotic reduction and sporulation. This will necessarily entail further studies with natural α/α diploid strains of one serotype (αAAα, αDDα, or αBBα), as the molecular differences are more subtle within each serotype, allowing meiosis. Detailed investigation of such isolates, as has been conducted for laboratory-generated αDDα hybrids [48], will provide insights on the complete same-sex mating cycle as it may occur in nature.

A common feature of the **a**ADα and αADα hybrid isolates is that they all bear a C-terminal deletion in the *SXI1Dα* gene. Selection for this allele likely occurred prior to the cell–cell fusion events that produced these hybrid strains. Because all of the **a**ADα and αADα hybrids tested bear the same truncated *SXI1Dα* allele whereas it is uncommon in haploid serotype D α isolates (∼13%), we favor the hypothesis that this allele enhances the fertility of Dα isolates. This interpretation is further supported by the observation that, unlike a complete deletion of the *SXI1α* gene, the C-terminally truncated *SXI1α* is still functional and Dα strains with this allele mate robustly and undergo meiosis normally. This hypothesis is also supported by the observation that C. gattii VGIII strains with a similar shortened version of *SXI1α* caused by a premature C-terminal stop codon also mate robustly. Because the VGIII group includes most of the fertile C. gattii strains characterized thus far, this shortened allele of *SXI1α* may also be associated with increased fertility. However, cell fusion between a transgenic strain with only the C-terminally truncated *SXI1Dα* allele in the JEC21 background and D**a** or A**a** partners was not enhanced compared to wild-type under the laboratory conditions tested thus far (data not shown). It is thus not clear if this truncated allele of *SXI1Dα* directly promotes the cell fusion step of mating, or is linked to another causative mutation in the *MAT* locus that was not detected in our study. Another possibility is that the effect of C-terminal truncation of *SXI1α* is genotype specific and mediated in concert with other unlinked mutations, similar to the observation that the role of the mating type locus in virulence is dependent on genetic background and functions as a quantitative trait locus [49,102]. Alternatively, laboratory conditions may not recapitulate the natural environment where cell fusion and mating occur (pH, temperature, nitrogen source, nutrient, and presence or absence of small molecules such as inositol and auxin indole-3-acetic acid [103]). The efficiency of cell fusion varies considerably depending on the isolate and mating medium (unpublished data). The last and, in our view, least likely possibility is that these alterations in the *SXI1α* gene are neutral variants, and by chance C-terminal truncation and the premature stop codon arose independently in the original ancestors of both the **a**ADα and αADα hybrid populations (the founder Dα strains) and the C. gattii VGIII strains. Our study demonstrates the complexity and diversity of the life cycles of C. neoformans and indicates that hybridization is influenced by both environmental and genetic factors.

Hybridization between two serotypes may have consequences for pathogenesis, as new strains with altered virulence may arise. The fact that AD hybrids occur at a reasonable frequency in both clinical and environmental samples is possibly indicative of hybrid fitness and an impact of hybridization on C. neoformans infection [33,35,61]. To test the effect of hybridization on virulence, yet avoid variations caused by natural genotypic differences and potential complications from the presence of both mating types, αADα hybrid strains were constructed in defined genetic backgrounds (H99 and JEC21), for which complete genome sequences are available and which are widely used in genetic and pathogenesis studies. The constructed αADα hybrid exhibited hybrid vigor under defined conditions, such as growth at high temperature (39 °C) and resistance to UV irradiation. The hybridization effect on melanization is complex and is affected by growth temperature. In most aspects tested in vitro, the αADα hybrid and Aα strains exhibited enhanced fitness compared to the less virulent Dα parental strain. Virulence tests in a murine inhalation model showed that the constructed αADα hybrid is similar in virulence to the Aα parental strain, while the Dα parental strain is almost avirulent. Overall, these observations support the hypothesis that hybridization between serotype A and D enhances the ability of the less virulent serotype D strains to survive both in the environment and in the host. Similar hybrid vigor (UV resistance and tolerance to high temperature) has also been observed in natural **a**ADα hybrids, and the increased fitness of these hybrids is hypothesized to have contributed to their worldwide distribution, whereas the parental A**a** strains are geographically restricted to Africa [59].

Our findings provide definitive evidence that C. neoformans can undergo same-sex mating in nature. However, a limitation is that natural αADα hybrids have an impaired ability to undergo meiosis and fail to produce haploid progeny, precluding further evaluation with these isolates of the impact of this life style on the haploid population structure and evolution of the C. neoformans species complex. The hybrid vigor displayed by the laboratory-constructed αADα strain, both in vitro and in vivo, offers a plausible explanation for the common presence of hybrids in clinical and environmental isolates. Whether AD hybrids are a source of diversity, are en route to speciation, or are a genetic dead end requires further investigation.

The unique α–α unisexual mating cycle that C. neoformans can adopt reflects either an adaptation to the sharply skewed distribution of mating types, or a route by which this disparity arose. It may maximize the advantages of both outcrossing and selfing in this heterothallic fungus that has a largely unisexual population. Similar strategies may also occur in other fungal species. For example, the obligate human fungal pathogen, Pneumocystis carinii, may share a similar life cycle. P. carinii is hypothesized to undergo both asexual and sexual cycles, based on cytological studies [104–106]. Only one mating-type-like region is known in this fungus, and there is no evidence of mating type switching [107]. The life cycle of the filamentous hemiascomycetous fungus Ashbya gossypii may also involve fusion of cells or nuclei of like mating type that then undergo meiosis and sporulation, as only the **a** allele of the *MAT* locus has been identified thus far for this species [18,108]. Similar inbreeding/selfing reproductive strategies have evolved in other kingdoms. For example, in plants that normally outcross, pseudo-self-compatibility in older flowers allows self-pollenization by a breakdown of self-incompatiblity barriers [109,110], which is conceptually similar to the ability of a heterothallic fungus to engage in same-sex mating. The first gene underlying pseudo-self-compatibility, the S-locus-linked gene *PUB8* (the S locus in plants is functionally similar to the mating type locus in fungi or the sex chromosomes in animals), was recently identified [111]. In many insects, parthenogenesis is also conceptually reminiscent of same-sex mating in fungi. Reproduction in parthenogenic strains of the bisexual species Drosophila mercatorum is also analogous to same-sex mating in the heterothallic species *C. neoformans* [112]. It has been shown in grasshoppers that parthenogenic species can generate a level of variability similar to that in closely related sexual species [113]. Similarly, same-sex mating in C. neoformans could potentially contribute significantly to genetic variation in the largely unisexual fungal population. Elucidating how this life cycle occurs in the genetically tractable fungus C. neoformans, its underlying molecular mechanisms, and its impact on population structure will shed light on similar reproductive strategies occurring in other species.

## Materials and Methods

### Strains and growth conditions.

The congenic strains JEC21 (α), JEC20 (**a**), H99, and KN99**a** were used as mating reference strains. Other strains used in this study are CHY621 (ura^−^, NAT^R^) [114], *sxi1*Δ mutants CHY610 [60] and CHY618 (ura^−^, NAT^R^) [114], XL1462 (αADα), XL1501 (αAAα), XL1620 (SXI1DαΔC), and those listed in Table 2. Cells were grown on YPD (1% yeast extract, 2% BactoPeptone, and 2% dextrose) or YNB medium (Difco). Mating or cell fusion was conducted on V8 medium (pH 7.0) in the dark at 22 °C.

### Determination of mating type.

To determine mating type, isolates were grown on YPD medium for 1 d at 30 °C and separately cocultured with the reference tester strains, JEC20 (*MAT* ***a***) and JEC21 (*MATα*), on V8 medium in the dark at 22 °C [78]. The isolate and tester strains were cultured alone on the same plate as controls. The mating reactions were examined after a week for mating hyphae formation, which signaled the initiation of sexual reproduction. Mating type was also determined by PCR with *SXI1α*, *SXI2* ***a***, and *STE20α/* ***a*** gene primers that yield mating-type- and serotype-specific amplicons. Primers used are listed in Table 3.

**Table 3:**
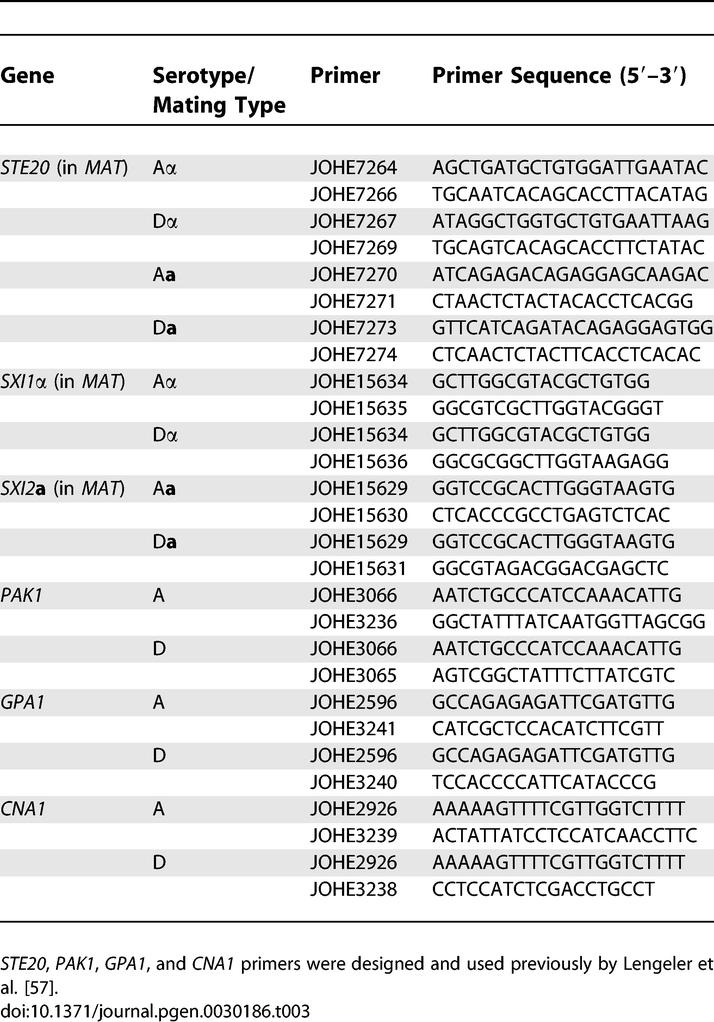
Serotype- and Mating-Type-Specific Primers

### Ploidy determination by fluorescence flow cytometry.

Cells were processed for flow cytometry as described previously [48,97]. Briefly, cells were harvested from YPD medium, washed once in PBS buffer, and fixed in 1 ml of 70% ethanol overnight at 4 °C. Fixed cells were washed once with 1 ml of NS buffer (10 mM Tris-HCl [pH 7.6], 250 mM Sucrose, 1 mM EDTA [pH 8.0], 1 mM MgCl_2_, 0.1 mM CaCl_2_, 0.1 mM ZnCl_2_) and then stained with propidium iodide (10 mg/ml) in 0.2 ml of NS buffer containing RNaseA (1 mg/ml) at 4 °C for 4–16 h. Then 0.05 ml of stained cells was diluted into 2 ml of 50 mM Tris-HCl (pH 8.0) and sonicated for 1 min. Flow cytometry was performed on 10,000 cells and analyzed on the FL1 channel with a Becton-Dickinson FACScan.

### Genomic DNA preparations.

Strains were grown in 50 ml of YPD medium at 30 °C overnight with shaking. The cells were washed three times with distilled water and harvested by centrifugation at 4,000*g* for 8 min. The cell pellet was frozen immediately at −80 °C, lyophilized overnight, and stored at −20 °C until genomic DNA was prepared using the CTAB protocol as described previously [115]. The quality of the purified DNA was examined on an agarose gel.

### Determination of molecular type by AFLP analysis.

AFLPs were generated and analyzed as previously described [53]. Two different EcoRI primer combinations (EAC and ETG) were used for the selective PCR, as described previously [68]. Only intense and reproducible bands were scored to identify differences between strains.

### Southern hybridization.

Genomic DNA was extracted as described above. DNA was digested with restriction enzymes, separated in agarose gels, and blotted to nitrocellulose (Zeta-Probe, Bio-Rad) by standard methods. Probes were generated with a Prime-It II kit (Amersham). Hybridization was performed using Ultrahyb (Ambion) according to the manufacturer's instructions.

### CGH and data analysis.

Genomic DNA was sonicated to generate ∼500-bp fragments and purified with a DNA Clean and Concentrator kit (Zymo Research). Five micrograms of DNA was used for Cy-3 dUTP or Cy-5 dUTP labeling reactions using the Random Primer/Reaction Buffer mix (BioPrime Array CGH Genomic Labeling System, Invitrogen). Hybridization conditions were as described previously [82] except that the slides contained a C. neoformans whole genome 70-mer oligonucleotide array and serotype- and mating-type-specific 70-mer oligonucleotides for genes in the *MAT* locus [49]. After hybridization, arrays were scanned with a GenePix 4000B scanner (Axon Instruments) and analyzed using GenePix Pro version 4.0 and BRB ArrayTools (developed by R. Simon and A. Peng Lam at the National Cancer Institute; http://linus.nci.nih.gov/BRB-ArrayTools.html).

### Microscopy.

Cells were grown on V8 medium in the dark at 22 °C. Hyphae were fixed in 3.7% formaldehyde and permeablized with 1% Triton in PBS. Nuclei were visualized by staining with DAPI (4′,6-diamidino-2-phenylindole, Sigma) as described previously [47].

### Size polymorphisms in the *SXI1Dα* gene.

PCR products of the *SXI1Dα* gene were generated using primers JOHE17409 (GCCGTGCAAGGGTGTAGG) and JOHE14895 (GGGCCATTGGAGGAAGCTG) and template genomic DNA from the strains tested. The PCR products were subjected to agarose gel electrophoresis to reveal different sizes of the *SXI1Dα* alleles in these strains.

### Construction of an αADα hybrid strain.

To construct the αADα hybrid strain, the auxotrophic strains F99 (Aα *ura5*) and XL342 (Dα *ade2*) were cocultured together with strain JEC169 (D**a** *ade2 lys1 ura5*) as the pheromone donor on V8 agar medium (pH 5.0) in the dark at 22 °C. These three strains are unable to grow on minimal medium without supplementation of uracil, adenine, and uracil + adenine + lysine, respectively. After 24 h of coculture on V8 medium, cells were collected and spread on YNB minimal medium at 37 °C to select for prototrophic fusion products. Two types of fusion products were obtained: the desired diploid αADα hybrid strains and triploid D**a**/Aα/Dα strains, which were distinguished by mating behavior and ploidy analyzed by flow cytometry analysis. The chosen diploid αADα hybrid strains were further confirmed by mating-type- and serotype-specific PCR analyses using primers listed in Table 3.

### In vitro assay of virulence factors.

Yeast cells were grown in YPD liquid medium overnight at 30 °C. Cells were collected by centrifugation and washed three times with sterile distilled water. Cell density was determined by absorption at 600 nm and cells were 10× serially diluted with sterile water. To examine melanin production, 3 μl of serial dilutions of cells were spotted on melanin-inducing medium containing L-DOPA (100 mg/l) [116] and incubated at 22 °C and 37 °C in the dark for 2 to 4 d. Melanization was observed as the colony developed a brown color. To analyze growth at different temperatures, cells were spotted on YPD medium and incubated at the indicated temperatures. Cell growth was assessed on days 2, 3, and 4. To determine sensitivity to UV irradiation, cells were spotted on YPD medium, air dried for 15 min, and then exposed to UV irradiation (∼48 mJ/cm^2^) in a Stratalinker (Stratagene) for 0, 6, or 12 s. Cells were then incubated at 22 °C, and cell growth was monitored daily from day 2 to 4. To characterize capsule production, equal numbers of C. neoformans cells were spotted on DMEM (Invitrogen) and incubated at 37 °C for 3 d. Cells were scraped from the plates, suspended in India ink, and observed microscopically. The capsule was visualized with light microscopy as a white halo surrounding the yeast cell due to exclusion of the dark ink particles.

### Mouse infection and recovery of fungal cells.

Mice were infected essentially as previously described [117]. Groups of 4- to 8-wk-old female A/J mice (ten mice per strain) were anesthetized by intraperitoneal injection of phenobarbital (∼0.035 mg/g). Animals were infected intranasally with 5 × 10^4^ fungal cells in 50 μl of PBS. The inocula of yeast cells were confirmed by CFU after serial dilutions. To verify strain identity for the inoculation, 100 colonies for the controls and 200 colonies for the hybrid were tested for auxotrophic markers and mating type. Three colonies for each strain were randomly chosen and checked for ploidy by fluorescent flow cytometry. Mice were monitored twice daily, and those showing signs of severe morbidity (weight loss, extension of the cerebral portion of the cranium, abnormal gait, paralysis, seizures, convulsions, or coma) were sacrificed by CO_2_ inhalation. The survival rates of animals were plotted against time, and *p-*values were calculated with the Mann–Whitney test. The lungs and brains from two animals from each group were removed, weighed, and homogenized in 2 ml of sterile PBS. Serial dilutions of the organ samples were plated on YPD agar plates containing 100 μg/ml chloramphenicol and incubated at 37 °C overnight. Randomly chosen colonies (100 for the controls and 200 for the hybrid) were tested for auxotrophic markers and mating type. Three colonies from each organ were randomly picked and checked for ploidy by fluorescent flow cytometry. Results of auxotrophic marker, mating type, and ploidy analysis of recovered strains were congruent with those for the infecting strains.

## Supporting Information

Figure S1 A**a** and D**a** *MAT* Locus Probes Are Functional Fragmented genomic DNA from strains H99 (Aα), KN99**a** (A**a**), JEC20 (D**a**), and JEC21 (Dα) was labeled with fluorescent dyes and competitively hybridized to a 70-mer genome array. The fluorescent signal level was normalized across the genome, and the average of at least two independent replicates of the fluorescent intensity ratio for the serotype A and D *MAT* locus **a** alleles is shown. “A” or “D” at the end of each gene name indicates the serotype A– or serotype D–specific allele. The schematic representation of the **a** mating type locus is illustrated at the bottom. Light brown indicates intergenic regions, red indicates the pheromone gene cluster, and white indicates highly conserved genes [9].
(42 KB PDF)

Figure S2 CGH of the Mating Type Locus of an Unrecognized αADα Hybrid Fragmented genomic DNA from isolate 713 and a mixture of genomic DNA from strains H99 (Aα) and JEC21 (Dα) was labeled with fluorescent dyes and competitively hybridized to a 70-mer genome array. The fluorescent signal level was normalized across the genome, and the average of three independent replicates of the fluorescent intensity ratio for the serotype A and D *MAT* locus α alleles is shown.
(31 KB PDF)

Figure S3 Multiple Alignment of the Predicted Amino Acid Sequences Encoded by *SXI1α* Genes The premature stop codon present in the majority of VGIII C. gattii strains is located in the C-terminal encoding region of the *SXI1α* gene, similar to the deletion site found in the *SXI1Dα* allele in the **a**ADα and αADα populations. The C-terminal truncated Sxi1α found in the αADα hybrid isolates is at the top, followed by the wild-type Sxi1α sequence of the serotype D strain JEC21, the wild-type Sxi1α sequence of the serotype A strain H99, the wild-type Sxi1α sequence of the C. gattii VGI strain WM276, and the translated sequences of Sxi1α of C. gattii VGII strain WM178, C. gattii VGIII strain V28, C. gattii VGIII strain DUMC140.97, C. gattii VGIII strain NIH312, C. gattii VGIII strain 97/426, C. gattii VGIII strain 97/433, C. gattii VGIII strain 97/428, C. gattii VGIII strain ICB88 (DQ198315), and C. gattii VGIII strain NIH836. Note that the VGIII strain NIH836 contains an early stop codon in the gene likely to render it nonfunctional. The sequences were aligned using GeneDoc (version 2.6.002) (http://www.nrbsc.org/gfx/genedoc/index.html) with default parameters.
(60 KB PDF)

Figure S4 A Transgenic Dα Strain Bearing the *SXI1Dα* C-Terminal Deletion in the JEC21 Background Is Fertile Wild-type strain JEC21, the *sxi1*Δ mutant CHY618 [6], and the transgenic *sxi1*Δ*C* strain XL1620 mated with A**a** reference strain KN99**a** on V8 medium (pH 7.0) in the dark at 22 °C for 2 wk. The upper row shows mating hyphae with basidiospores by microscopy (×200). The insets are the mating colonies. The lower row shows the cell fusion assay for the corresponding mating as described above after 24 h of coincubation on V8 medium. Cells were collected from V8 medium, and the fusion products were grown on selective medium at 37 °C for 3 d (see Text S1 for details).
(17.8 MB TIF)

Figure S5 αADα Hybrid Is More Resistant to UV Irradiation Than Isogenic Haploid or Diploid Isolates Aα strain H99, αAAα strain XL1501, Dα strain JEC21, αDDα strain XL143, and αADα strain XL1462 were grown in liquid YPD medium overnight and washed three times with distilled water. Cell concentration was determined by counting with hemacytometer. Three microliters of serial dilutions (10×) of cells at the same cell density were spotted onto YPD medium at 22 °C as controls for growth (first column). Cells on YPD medium were subjected to UV irradiation for 10 s or 15 s (∼48 mJ/cm^2^) and then incubated at 22 °C in the dark (second and third columns, respectively).
(7.0 MB TIF)

Text S1 Supplemental Methods and Materials (30 KB DOC)

### Accession Numbers

The GenBank (http://www.ncbi.nlm.nih.gov/Genbank/index.html) accession numbers for the Sxi1α sequences discussed in this paper are as follows: C. neoformans αADα hybrid (EF471284), C. neoformans wild-type strain JEC21 (AAN75718), C. neoformans wild-type strain H99 (AAN75175), C. gattii wild-type VGI strain WM276 (AAV28797), C. gattii VGII strain WM178 (DQ096309), C. gattii VGIII strain V28 (AY973651), C. gattii VGIII strain DUMC140.97 (DQ096306), C. gattii VGIII strain NIH312 (DQ096307), C. gattii VGIII strain 97/426 (DQ198312), C. gattii VGIII strain 97/433 (DQ198313), C. gattii VGIII strain 97/428 (DQ198314), C. gattii VGIII strain ICB88 (DQ198315), and C. gattii VGIII strain NIH836 (DQ198305).

## Abbreviations

AFLP: amplified fragment length polymorphism
CGH: comparative genome hybridization
L-DOPA: L-dihydroxyphenylalanine
*SXI1D*α: *SXI1*α serotype D
