# Supplementary material for: αADα Hybrids of Cryptococcus neoformans: Evidence of Same-Sex Mating in Nature and Hybrid Fitness
Source: PLoS Genet. 2007 Oct 19;3(10):e186. doi: 10.1371/journal.pgen.0030186 (PMC2042000; doi:10.1371/journal.pgen.0030186)

*MAT**a*** probes are functional

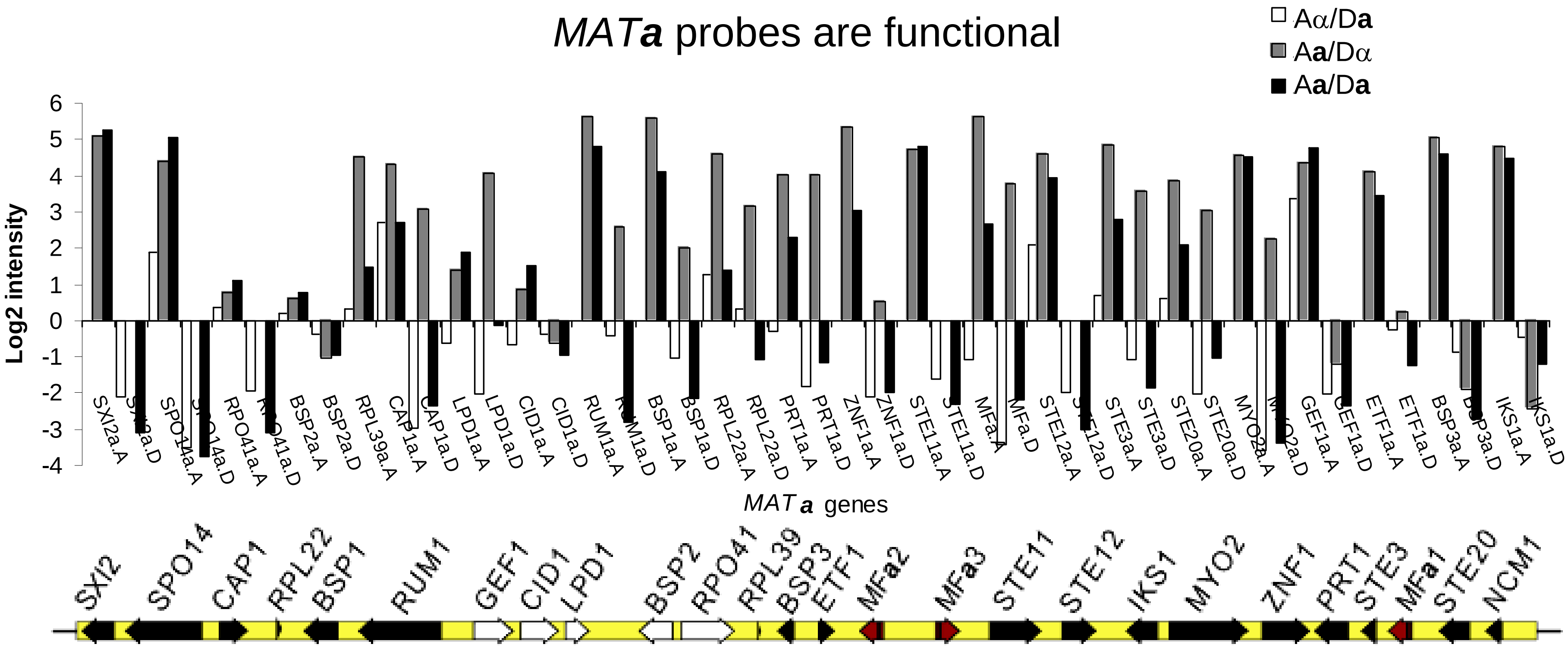

Supplement: Supplementary file 1 [file pgen.0030186.sg001.pdf]
