# Supplementary material for: αADα Hybrids of Cryptococcus neoformans: Evidence of Same-Sex Mating in Nature and Hybrid Fitness
Source: PLoS Genet. 2007 Oct 19;3(10):e186. doi: 10.1371/journal.pgen.0030186 (PMC2042000; doi:10.1371/journal.pgen.0030186)
Supplement: Supplementary file 3 [file pgen.0030186.sg003.pdf]

```
                     *        20         *        40         *        60         *        80         *       100         *       120         *       140
Sxi1-Del   : MLSTCDSSSQSIRRSLQDDLLDFQETFAKALGKREVHDLESVVQKGNKLVTAIKWSSSRDLLDKNTASLAYAVASSVKLIGSSALELEGGREDAIGEAMLRIESLLLDEGQSRKRQQRNHVPRLKRYCRGSSSESSIAWP : 140
D-AAN75718 : MLSTCDSSSQSIRRSLQDDLLDFQETFAKALGKREVHDLESVVQKGNKLVTAIKWSSSRDLLDKNTASLAYAVASSVKLIGSSALELEGGREDAIGEAMLRIESLLLDEGQSRKRQQRNHVPRLKRYCRGSSSESSIAWP : 140
A-AAN75175 : MLSTSDSSSQSIQRSLQDDLLDFQETFTQPLGKSEVHDLESVVRKGSNLVTAIKWSSSHDLLDENTASLAYAVARSVKLIGSSALKLEQGWEDAVGEAILRIESLLPDEGQSRKRRQRNHEPRPKRYCRGPSSESCLAWP : 140
WM276-AAV2 : MLSTCDTSSQSIRKSLQDDLVDFQETFTKVLGKREIDGHESVARKGSNLVTAVKWSSSRHLLDENTVSLAYAVAHNVKVIGSSALQLEQGREDAVGEAMLRIESLLLDQGRRRKCRQRDYEPQPKRCCRRSSSESYNTWP : 140
DQ096309   : MLSTCDTSSQSIRKSLQDDLVDFQETFAKILGKRKIDDNENVARKWRNLVTAVKWSSSRHLLDENTVSLVYAVAHNVKVIGSSALQLERGRENAVGDAMLRIESLLLDQGRRRKCHQRDYEPQPKRCCCGSSSESYSTWP : 140
AY973651   : MLSTCDTSSQSIRKSLRDDLVDFQETFAKVLGKSEIDDHENVTRKGRNLVTAVKWSSSPHLLDENTVSLAYAVAHNVKVIGSSALQLERGREDAVGEAMLRIESLLLDQGRRRKCRQRNYEPQPKRCCRGSSSESYSTWP : 140
DQ096306   : MLSTCDTSSQSIRKSLRDDLVDFQETFAKVLGKSEIDDHENVTRKGRNLVTAVKWSSSPHLLDENTVSLAYAVAHNVKVIGSSALQLERGREDAVGEAMLRIESLLLDQGRRRKCRQRNYEPQPKRCCRGSSSESYSTWP : 140
DQ096307   : MLSTCDTSSQSIRKSLRDDLVDFQETFAKVLGKSEIDDHENVTRKGRNLVTAVKWSSSPHLLDENTVSLAYAVAHNVKVIGSSALQLERGREDAVGEAMLRIESLLLDQGRRRKCRQRNYEPQPKRCCRGSSSESYSTWP : 140
DQ198312   : MLSTCDTSSQSIRKSLRDDLVDFQETFAKVLGKSEIDDHENVTRKGRNLVTAVKWSSSPHLLDENTVSLAYAVAHNVKVIGSSALQLERGREDAVGEAMLRIESLLLDQGRRRKCRQRNYEPQPKRCCRGSSSESYSTWP : 140
DQ198313   : MLSTCDTSSQSIRKSLRDDLVDFQETFAKVLGKSEIDDHENVTRKGRNLVTAVKWSSSPHLLDENTVSLAYAVAHNVKVIGSSALQLERGREDAVGEAMLRIESLLLDQGRRRKCRQRNYEPQPKRCCRGSSSESYSTWP : 140
DQ198314   : MLSTCDTSSQSIRKSLRDDLVDFQETFAKVLGKSEIDDHENVTRKGRNLVTAVKWSSSPHLLDENTVSLAYAVAHNVKVIGSSALQLERGREDAVGEAMLRIESLLLDQGRRRKCRQRNYEPQPKRCCRGSSSESYSTWP : 140
DQ198315   : MLSTCDTSSQSIRKSLRDDLVDFQETFAKILGKSEIDDHENVTRKGRNLVTAVKWSSSPHLLDENTVSLAYAVAHNVKVIGSSALQLERGREDAVGEAMLRIESLLLDQGRRRKCRQRNYEPQPKRCCRGSSSESYSTWP : 140
DQ198305   : MLSTCDTSSQSIRKSLRDDLVDFQETFAKVLGKSEIDDHENVTRKGRNLVTAVKWSSSPHLLDENTVSLAYAVAHNVKVIGSSALQLERGREDAVGEAMLRIESLLLDQGRRRKCRQ----------------------- : 117

                     *       160         *       180         *       200         *       220         *       240         *       260         *       280
Sxi1-Del   : KSTFDNSPKPSEPPIDHTVVRLWFLDNLAYPYPTAQQKDFLAKTAGIQRSQVDSDLTNYRRRAGWTDIMNVWCGGDRNAMKKLMNRVEKGKEQRKKILDAVQGCRDYLTMKENNKVGDWIKEITQNSAFKYRFTGS-LPD : 279
D-AAN75718 : KSTFDNSPKPSEPPIDHTVVRLWFLDNLAYPYPTAQQKDFLAKTAGIQRSQVDSDLTNYRRRAGWTDIMNVWCGGDRNAMKKLMNRVEKGKEQRKKILDAVQGCRDYLTMKENNKVGDWIKEITQNSAFKYRFTGS-LPD : 279
A-AAN75175 : ESTFGNSPKPSESATDHTVVRLWFLDNLAYPYPTAQQKDFLAKTAGIQRSQVDSDLTNYRRRAGWTDIMNMWCGGDRSAMKKLMERVERGKEQRKKILDAVQGCRDYLTMKENNKIGDWVKEITQNSTSKYRFTGF-LPD : 279
WM276-AAV2 : ESNLHISPKPLESAPDHTIVRLWFLNNLAYPYPTAQQKDSLAKYAGMQRGKVDSDLTNYRRRAGWTDIMNMWCGGDRNAMKKLMEKVERGKEQRKEILDAVQGCKDYLTMKESNRVGDWVKEITQNSDSKYCFGGIILPN : 280
DQ096309   : ESNLHISPKPSESAPDHTIVRLWFLNNLAYPYPTAQQKESLAKHARMQRSKVDSDLTNYRRRAGWTDIMNVWCGGDRNAMKKLMEKVERGKEQRKEILDAVQGCKDYLTMKENNRVGDWVKEITRNSDSKYRFTGILPMC : 280
AY973651   : ESNLHISPKPSESAPDHTIVRLWFLNNLAYPYPTTQQKDSLAKYAGMQRSKIDSDLTNYRRRAGWTDIMNVWCGGDRNAMKKLMEKVERGKEQRKEILDAVQRCKDYLTMKENNRVGDWVKEITQNSGSKYRFTGIILPN : 280
DQ096306   : ESNLHISPKPSESAPDHTIVRLWFLNNLAYPYPTTQQKDSLAKYAGMQRSKIDSDLTNYRRRAGWTDIMNVWCGGNRNAMKKLMEKVERGKEQRKEILDAVQRCKDYLTMKENNRVGDWVKEITQNSGSKYRFTGIILPN : 280
DQ096307   : ESNLHISPKPSESAPDHTIVRLWFLNNLAYPYPTTQQKDSLAKYAGMQRSKIDSDLTNYRRRAGWTDIMNVWCGGNRNAMKKLMEKVERGKEQRKEILDAVQRCKDYLTMKENNRVGDWVKEITQNSGSKYRFTGIILPN : 280
DQ198312   : ESNLHISPKPSESAPDHTIVRLWFLNNLAYPYPTTQQKDSLAKYAGMQRSKIDSDLTNYRRRAGWTDIMNVWCGGNRNAMKKLMEKVERGKEQRKGILDAVQRCKDYLTMKENNRVGDWVKEITQNSGSKYCFTGIILPN : 280
DQ198313   : ESNLHISPKPSESAPDHTIVRLWFLNNLAYPYPTTQQKDSLAKYAGMQRSKIDSDLTNYRRRAGWTDIMNVWCGGDRNAMKKLMEKVERGKEQRKEILDAVQRCKDYLTMKENNRVGDWVKEITQNSGSKYRFTGIILPN : 280
DQ198314   : ESNLHISPKPSESAPDHTIVRLWFLNNLAYPYPTTQQKDSLAKYAGMQRSKIDSDLTNYRRRAGWTDIMNVWCGGDRNAMKKLMEKVERGKEQRKEILDAVQRCKDYLTMRENNRVGDWVKEITQNSGSKYRFTGIILPN : 280
DQ198315   : ESNLHISPKPSESAPDHTIVRLWFLNNLAYPYPTTQQKDSLAKYAGMQRSKIDSDLTNYRRRAGWTDIMNVWCGGDRNAMKKLMEKVERGKEQRKEILDAVQRCKDYLTMKENNRVGDWVNEITQNSGSKYRFTGIILPN : 280
DQ198305   : -------------------------------------------------------------------------------------------------------------------------------------------- :   -

                     *       300         *       320         *       340         *       360         *       380         *       400         *
Sxi1-Del   : ISAASPCSSIRPCTPRSFSGSSATSSTSSCSEVSEASAIITIPRKRRYTGDESISPFKRVQGQTVEVSGSYEPWSFADLQPMPPSP---------------------------------------CEGLI : 370
D-AAN75718 : ISAASPCSSIRPCTPRSFSGSSATSSTSSCSEVSEASAIITIPRKRRYTGDESISPFKRVQGQTVEVSGSYEPWSFADLQPMPPSPGAAQEDIDNEFVAPTTYFISATSLSLSSPAEPLTKPRLAC---- : 405
A-AAN75175 : MSAASPCSGIRPSTPRSFSGSSAISSMSSCSEVSEASAIIPIPRKRCYTDDEPISPLKRARNHTVGVSGPYESWSFADLQPMPPSPGAAQENTANDSVAPTTYFISAASRSLPSSAETRTKRRLSC---- : 405
WM276-AAV2 : VSVASPYSTNRFSTARSFSGSSALSSMSSCSEVSEASTIIPISSKKRYTRDESISPFKRTRNQTAEVSGSYEPWSIADLQPMPPSPGAAEENTANDSIVPATCLVNPSPLSLSPPGETRTKPRPAC---- : 406
DQ096309   : PSPLHALPINAALLVPSPVVVPSLACLLAPKSLRLLQSSLFLVRNVTPATSPFLPSNALETRLLRSLVLMSLGPSPTYSLCLLLPVQPKRTPLTILSSPQPASSMPPLSHYLHLAKLVPSRG-------- : 402
AY973651   : VTVASPCSTNRCSTARSFSGSSALSSMSSCSEVSEASAIIPIPSKKRYTRDESISPFKRTRNQTAEVSGSYEPWSIADL--------------------------------------------------- : 359
DQ096306   : VTVASPCSTNRCSTARSFSGSSALSSMSSCSEVSEASAIIPIPSKKRYTRDESISPFKRTRNQTAEVSGSYEPWSIADL--------------------------------------------------- : 359
DQ096307   : VTVASPCSTNRCSTARSFSGSSALSSMSSCSEVSEASAIIPIPSKKRYTRDESISPFKRTRNQTAEVSGSYEPWSIADL--------------------------------------------------- : 359
DQ198312   : VTVASPCSTNRCSTARSFSGSSALSSMSSCSEVSEASAIIPIPSKKRYTRDESISPFKRTRNQTAEVSGSYEPWSIADL--------------------------------------------------- : 359
DQ198313   : VTVASPCSTNRCSTARSFSGSSALSSMSSCSEVSEASAIIPIPSKKRYTRDESISPFKRTRNQTAEVSGSYEPWSIADL--------------------------------------------------- : 359
DQ198314   : VTVASPCSTNRCSTARSFSGSSALSSMSSCSEVSEASAIIPIPSKKRYTRDASISPFKRTRNQTAEVSGSYEPWSIADL--------------------------------------------------- : 359
DQ198315   : VTVASACSTNRCSTARSFSGSSALSSMSSCSEVSEASAIMPIPSKKRYTRDESISPFKRTRNQTAEVSGSYEPWSIADL--------------------------------------------------- : 359
DQ198305   : ---------------------------------------------------------------------------------------------------------------------------------- :   -
```
