# Supplementary material for: αADα Hybrids of Cryptococcus neoformans: Evidence of Same-Sex Mating in Nature and Hybrid Fitness
Source: PLoS Genet. 2007 Oct 19;3(10):e186. doi: 10.1371/journal.pgen.0030186 (PMC2042000; doi:10.1371/journal.pgen.0030186)
Supplement: Supplementary file 4 [file pgen.0030186.sd001.doc]

**Supplemental methods and materials**

*Generation of sxi1ΔC strain in JEC21 background.* To generate a C-terminally truncated *SXI1Dα* strain in the JEC21 background, an overlap PCR product with the NAT marker and the C-terminal truncated version of the *SXI1Dα*  locus from strain 431 was generated using overlap PCR and primers JH17409 (GCCGTGCAAGGGTGTAGG), JH17895 (CTGGCCGTCGTTTTACGCAAAGGGATACAGCCGAG), JH18406 (GTCATAGCTGTTTCCTGCCCTTCTTCAACTGCCCTTAC), JH18407(GGGCTTTTCATGATGTAGGG), M13 forward (GTAAAACGACGGCCAG), and M13 reverse (CAGGAAACAGCTATGAC). The overlap PCR product was directly introduced into strain JEC21 by biolistic transformation using a Bio-Rad Model PDS-1000/He Biolistic Particle Delivery System, as previously described [1]. Transformants were selected on YPD medium with NAT (100µg/ml). The insertion of the C-terminal truncated *SXI1Dα* allele at the native *SXI1Dα* locus was confirmed by PCR analysis to confirm the presence of C-terminal deletion in the *SXI1Dα* gene {primers JOHE17410 (CCTATGCCTCCTTCTCCC) and JOHE14895 (GGGCCATTGGAGGAAGCTG)}, and the absence of the native *SXI1Dα* gene (primers JOHE15634 and JOHE15636).

*Cell-fusion assay.* Wild type CHY621 (ura^-^, NAT^R^), *sxi1Δ* mutant CHY618 (ura^-^, NAT^R^) [2], transgenic *sxi1ΔC* strain XL1620 (ura^-^, NAT^R^), and A**a** reference strain KN99**a** (Ura^+^, nat^s^) were grown in YPD liquid medium overnight. Cells were washed three times with sterile water and quantified by hemacytometer to 10^8^ cells/ml. Equal number of cells for the mating partners were mixed together and 10 µl of the mating mixture were spotted on V8 plates (pH 7.0) at 22°C in the dark. After 24 hours of coincubation, cells were scraped from the V8 plates, resuspended in 500 µl of water and 200 µl of the suspension was spread on selective medium that supports growth of fusion product (Ura^+^, NAT^R^ ) as previously described [2].

*Construction of an αAAα diploid strain.* To construct an αAAα diploid strain, two strains YSB119 (Aα *aca1*::NAT^R^ *ura5* *ACA1-URA5*) [3] and KN99αNEO1 (Aα G418^r^) marked with dominant drug resistance markers were cocultured together with JEC156 (D**a** *ade2 ura5*) as pheromone donor on V8 (pH 5.0) + 50 µM CuSO_4_ medium in the dark at 22°C. Addition of copper in the medium enhances mating or fruiting of *C. neoformans* [4]. After 24 hours of coculture on V8 medium, cells were collected and spread on YPD+NAT+G418 medium at 37°C to select for fusion products. Two types of fusion products were obtained: the desired diploid αAAα diploid strains and triploid D**a**/Aα/Aα strains, which were distinguished by mating behavior and ploidy analysis by FACs. The chosen diploid αAAα hybrid strain XL1501 was further confirmed by mating type- and serotype-specific PCR analyses.

References

1. Toffaletti DL, Rude TH, Johnston SA, Durack DT, Perfect JR (1993) Gene transfer in *Cryptococcus neoformans* by use of biolistic delivery of DNA. J Bacteriol 175: 1405-1411.

2. Hull CM, Boily MJ, Heitman J (2005) Sex-specific homeodomain proteins Sxi1alpha and Sxi2a coordinately regulate sexual development in *Cryptococcus neoformans*. Eukaryot Cell 4: 526-535.

3. Bahn YS, Cox GM, Perfect JR, Heitman J (2005) Carbonic anhydrase and CO_2_ sensing during *Cryptococcus neoformans* growth, differentiation, and virulence. Curr Biol 15: 2013-2020.

4. Lin X, Huang JC, Mitchell TG, Heitman J (2006) Virulence attributes and hyphal growth of *Cryptococcus neoformans* are quantitative traits and the *MATα* allele enhances filamentation. PLoS Genet 2: e187 (181-114).
